# COVID-19-associated cytokine storm syndrome and diagnostic principles: an old and new Issue

**DOI:** 10.1080/22221751.2021.1884503

**Published:** 2021-02-18

**Authors:** Xi Yongzhi

**Affiliations:** Department of Immunology and National Center for Biomedicine Analysis; Fifth Medical Center of Chinese PLA General Hospital, Beijing, People’s Republic of China

**Keywords:** SARS-CoV-2, COVID-19, cytokine storm syndrome, familial/primary hemophagocytic lymphohistiocytosis, secondary hemophagocytic lymphohistiocytosis

## Abstract

SARS-CoV-2 has claimed 2,137,908 lives in more than a year. Some COVID-19 patients experience sudden and rapid deterioration with the onset of fatal cytokine storm syndrome (CSS), which have increased interest in CSS’s mechanisms, diagnosis and therapy. Although the prototypic concept of CSS was first proposed 116 years ago, we have only begun to study and understand CSS for less than 30 years. Actually, diseases under CSS umbrella include familial/primary and secondary hemophagocytic lymphohistiocytosis (HLH), macrophage activation syndrome (MAS), infection-associated hemophagocytic syndrome, cytokine release syndrome (CRS), and cytokine storm (CS). Hematologic malignancies and autoimmune diseases that cause CSS are named malignancy-associated hemophagocytic syndrome (MAHS) and MAS, respectively. In-depth research on the pathogenesis of HLH/CSS has greatly increased the number of patients that were able to be definitively diagnosed with HLH/CSS. However, it should be emphasized that HLH/CSS diagnosis is difficult at the early stages due to the non-specific clinical signs and symptoms, which tends to result in missed and incorrect diagnoses. Therefore, clinicians should not only possess extensive clinical experience to ensure high sensitivity to the characteristics of HLH/CSS but must also be familiar with HLH-2004/2009 diagnostic criteria, and HScore methods. The paper concisely comment evolution of CSS classifications, cytokines associated with CSS, evolution of CSS diagnostic criteria and importance of the correct identification of hemophagocytes in diagnosing CSS, which is timely and may benefit clinicians familiar HLH-2004/2009 diagnostic criteria, and HScore methods. In addition, clinicians must also understand that there are some limitations to these diagnostic criteria.

Abbreviations: aBMT: autologous bone marrow transplantation; CAR-T: chimeric antigen receptor-engineered T-cell; COVID-19: coronavirus disease 2019; CSS: cytokine storm syndrome; HLH: hemophagocytic lymphohistiocytosis; MAS: macrophage activation syndrome; CRS: cytokine release syndrome; CS: cytokine storm; MAHS: malignancy-associated hemophagocytic syndrome; IAHS: infection-associated hemophagocytic syndrome; fHLH/pHLH: familial/primary hemophagocytic lymphohistiocytosis; sHLH: secondary hemophagocytic lymphohistiocytosis; SARS-CoV-2: severe acute respiratory syndrome coronavirus 2; TCR-T, T-cell receptor-engineered T-cell

## Introduction

Although China with the power of the whole country has passed the “Darkest Hour” of the COVID-19 pandemic, this disease has spread throughout the world, and the situation in the United States of America, Europe, Brazil, Russia and India is dire, akin to “The Dark Ages.” As of 25 January 2021, the number of confirmed COVID-19 cases worldwide reached 99,734, 823 resulting in more than 2,137,908 deaths [1]. The mortality rate is several times that of seasonal flu and far exceeds that of a major seasonal flu epidemic. At present, our greatest challenge is that our understanding of both SARS-CoV-2 and COVID-19, is still in Jahiliyyah. The fatal cytokine storm syndrome (CSS) that occurred in some patients who have died from COVID-19 is particular concern [2,3]. CSS has peaked interest not only in the medical world but also among various parties in society, and the number of indexed CSS papers has also increased [4,5,6,7,8,9,10,11,12]. However, the CSS observed in COVID-19 patients is not a new phenomenon. CSS is common not only in microbial and viral infections but also in autoimmune diseases, hematological malignancies, and after some biological treatments [2,3,13,14]. The aim of this paper was to review the various classifications of CSS, the associated major cytokines, and the progress in the international diagnostic criteria for CSS.

## Evolution of CSS classifications

Strictly speaking, CSS is not a new syndrome, as the rudimentary idea was first proposed 116 years ago. However, research on and our overall understanding of CSS only began in the last 30 years. Many clinicians, especially for senior physicians, have never even heard of CSS, although the fundamental feature of CSS, sepsis, is more broadly understood. Sepsis is an umbrella term that encompasses different aspects of CSS, and accumulated basic and clinical studies have shown that CSS is an excessive immune response that is caused not by a single disease, but by different etiologies, that results in hyperinflammation. CSS includes formerly used terms such as hemophagocytic lymphohistiocytosis (HLH), macrophage activation syndrome (MAS), infection-associated hemophagocytic syndrome(IAHS), cytokine release syndrome (CRS), and cytokine storm (CS). CSS was previously known as fHLH/pHLH and sHLH, both of which are caused by infections with pathogenic microorganisms. fHLH/pHLH was also known previously by different names, including familial reticulosis, lymphohistiocytic reticulosis, familial erythrophagocytic lymphohistiocytosis, familial histiocytosis, and generalized lymphohistiocytic infiltration and others. It was only after a review published in 1983 that fHLH/pHLH became the more widely used term. MAHS and MAS refer to CSS caused by haematologic malignancies and autoimmune disease, respectively. CSS that occurs during treatment with chimeric antigen receptor-engineered T-cell (CAR-T)/T-cell receptor-engineered T-cell (TCR-T) therapy or monoclonal antibody therapy is known as CRS, while CSS that occurs in autologous bone marrow transplantation (aBMT) is known as CS.

It is generally acknowledged in the literature that the true concept of CSS was proposed in 1993 when Ferrara and coworkers proposed CS while describing GVHD. But back in 1989, Chatenoud and coworkers discovered and reported CRS when using OKT3 anti-T-cell monoclonal antibodies to prevent kidney transplant rejection. However, neither CS nor CRS were the earliest described forms of CSS. The earliest rudimentary concept of CSS, sepsis, can be traced back to 1904, when it was described in the representative work “The Evolution of Modern Medicine” by Sir William Osler, who is known as the father of modern medicine [14]. In 1918, actually, CSS was found to be the major cause of death in a large number of young adults infected with the Spanish flu. In 1939, Scott and Robb-Smith firstly described a few clinical cases of histiocytic medullary reticulosis in the Lancet, which was later classified as fHLH/pHLH by Farquhar & Claireaux (1952). It since has been confirmed that fHLH/pHLH is an autosomal recessive monogenic disease that can be further classified as fHLH/pHLH and three types of immunodeficiency-related hemophagocytic lymphohistiocytosis (iHLH/pHLH), according to the pathogenic gene involved. The first report of sHLH caused by viral infection was as late as 1979, and it was termed virus-associated hemophagocytic syndrome by Risdall and coworkers. Subsequently, a lots of types of bacteria, protozoa, fungi, and viruses have been found to cause sHLH/CSS. Pathogenic microorganisms, particularly viruses, were found to be the cause of most CSS cases. IAHS/CSS can occur at any age, and adult IAHS/CSS accounts for 50% of adult sHLH/CSS cases [14,15,16,17,18,19]. During the 2002 SARS outbreak, it was found that SARS-CoV-1 infection can induce the production of high levels of interferon gamma (IFN-γ), resulting in CSS, ARDS, and MODS, leading to a mortality rate of 10%. This led to a new understanding of CSS by the medical world. In 2006, Henter and coworkers found that patients infected with H5N1 virus presented with increased peripheral blood cytokines, pancytopenia, and acute encephalitis, and targeted therapy for sHLH/CSS can significantly increased survival. In 2013, acute phase serum samples collected from patients infected with H7N9 virus were found to have elevated levels of numerous cytokines. After that, CSS was also identified in patients who had severe cases of MERS and Ebola. In these patients, it was CSS and not the virus itself that caused acute lung injury; CSS is considered to be one of the major factors behind the high mortality rate in patients with severe illness [16,17,18,19]. However, published studies, diagnostic guidelines, and treatment guidelines have mostly focused on pediatric HLH/CSS, and there are few epidemiological and clinical studies on adult CSS.

Surprisingly, contrary to the previous understanding of other diseases-associated CSS, we do have a better understanding of virological, epidemiological and clinical aspects of COVID-19-associated CSS just one year after SARS-CoV-2 pandemics. COVID-19-associated CSS indeed has its own some unique epidemiological characteristics except that clinical symptoms and signs (fever, generalised malaise, cough, stuffy nose, sputum, shortness of breath, and faster respiratory rates) are not specific and the same as other diseases-associated CSS. Different from other diseases-associated CSS, the incidence rate of COVID-19-associated CSS not only has sex and age disparity, but also ethnicity and comorbidities disparity, etc [2,20,21,22,23,24]. First, men appear to more susceptible to COVID-19-associated CSS in comparison with women although SARS-CoV-2 infection rate seems to be similar in both sexes. The reasons are that estrogens can make women’s a more effective innate and adaptive immune response and a less pronounced hyperinflammation. Besides, estrogens can effectively prevent viral entry and reduce viral load[20]. Second, COVID-19 patients are mainly middle-aged and elderly people, which is completely different from the 2003 SARS-CoV-1 epidemic, in which the main population affected was young adults. In particular, the elderly population is a high-risk group affected by SARS-CoV-2 pandemic due to the combined effects of aging, decreased immune function, poor nutrition, and comorbidity of high-risk chronic diseases. These show that the middle-aged and elderly people are not only more susceptible to SARS-COV-2 infection but also have the highest mortality rate among all age groups [21,22]. Third, some recent studies have showed that there are indeed racial differences especially amongst the Black and Minority Ethnic (BAME) population in incidence rate of COVID-19-associated CSS because BAME populations have the genetic predisposition to higher risks of metabolic syndrome. Metabolic syndrome has been proved as an inflammatory condition which have been associated with pro-inflammatory cytokine dysregulation of COVID-19 severity [23]. Fourth, there are accumulating evidence that patients with previous chronic viral or bacterial infections such as human Immunodeficiency virus, chronic hepatitis B or C, and tuberculosis are likely to develop COVID-19-associated CSS. This is because these infections are all associated with a chronic elevation in pro-inflammatory cytokines, which lower the threshold for triggering a “cytokine storm” after SARS-CoV-2 infection [23]. Fifth, There have been recent multiple reports providing further insight into the clinical course of COVID-19-associated CSS in which clinical course of each patient with COVID-19-associated CSS is changeable and irregular, which may result from quite different SARS-CoV-2 load, medical history, immune response, and cytokine release level, ect. [21,22]. Sixth, SARS-COV-2 infection also obviously lead to haematological alterations especially in patients with COVID-19. Some recent studies have indicated that 83.2% of COVID-19 patients presented with lymphocytopenia, 36.2% with thrombocytopenia, and 33.7% with leukopenia. COVID-19-associated CSS usually had more obvious leukopenia, thrombocytopenia and lymphocytopenia especially CD4^+^ T and CD8^+^ T cells, which leading to lowered IFN-γ production, All of these were identified as an independent predictor of more severe and critical COVID-19 [24]. Last, continuous discovery of pathogenic genes revealed that ∼10% of the general population carry mutations in CSS-susceptibility genes. Thus, the distinction between pHLH/CSS and sHLH/CSS is increasingly vague. Accumulating data have indicated that approximately 5–10% of COVID-19 patients have CSS whether this is a coincidence remains unknown. To allow early establishment of preventive and therapeutic strategies, COVID-19 patients should be screened for such mutations before the onset of CSS. This will help elucidate the pathogenesis of CSS and help develop efficient diagnostic and preventive protocols[2,23].

## Cytokines associated with CSS

**Figure 1.**
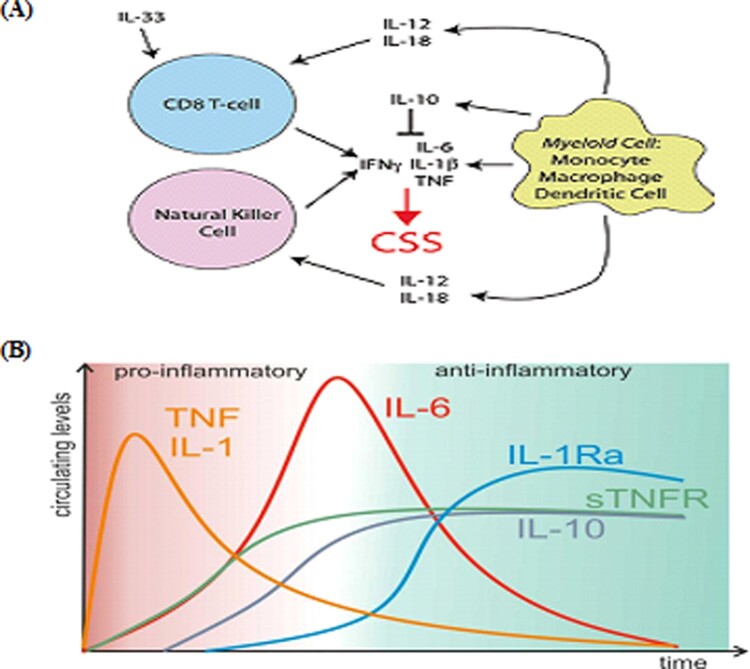
Schematic of cytokine networks(A) and cytokine kinetics (B) in cytokine storm syndrome. Accumulated studies have shown that IL-1β and TNF-α can promote the secretion of other cytokines such as IL-6 etc., and are thus known as early phase cytokines. After stimulation by pathogenic factors, IL-1β and TNF-α are rapidly secreted, peaking after several hours. Subsequently, the body starts to secrete anti-inflammatory cytokines to regulate inflammatory responses and enable the body to counteract stimulation induced by harmful pathogens and maintain cellular homeostasis. However, when there is continuous, strong stimulation from harmful pathogens or excessive immune responses, the equilibrium between pro- and anti-inflammatory responses is disrupted. Figure adapted from Refs. [14] and [29].

The pathogenesis of CSS is complex and diverse and differs among the various etiologies. Even the same etiology can result in different CSS pathogeneses. The inherent variance in CSS etiology leads to different pathophysiological changes in immune function that ultimately have the same end, and the core effects are intimately associated with NK and CTL-mediated cytotoxicity [13,14,15]^.^ On the one hand pathogenic microorganisms elicit excessive proliferation and activation of T-cells and macrophages, resulting in dysregulated cellular immunity and Th1/Th2 ratios, excessive Th1 cell activation, secretion of large amounts of cytokines that activate CTLs and macrophages, proliferation of a large number of CTLs, and enhanced phagocytosis in macrophages. Furthermore, these pathogenic microorganisms activate macrophages, DCs, NK cells, and CTLs, leading to a cytokine storm that damages target cells. As the infection ends and the target cells die, immune activation is terminated. If immune cell activation is not terminated, CTLs will be continuously activated by proliferation signals and secrete large amounts of IFN-γ, which in turn will persistently induce macrophages to secrete various cytokines and chemokines, resulting in a cascade of uninhibited inflammatory responses(Figure 1(A)) [13,14,15,16,17,18].

Cytokines are a class of small soluble polypeptides, and more than 200 have been discovered. Cytokines can be classified into six major classes according to their structure and function: interleukins (ILs), colony-stimulating factors (CSFs), IFNs, tumour necrosis factor (TNFs), growth factors (GF), and chemokines (CHs) [25,26,27,28]. Cytokines usually exert their effects through paracrine signaling on neighbouring cells or autocrine signalling on the cytokine-secreting cell. Some cytokines can bind to intracellular receptors for intracrine signalling. Alternatively, cytokine/cytokine receptor complexes can be internalized to exert their effects in cells. In juxtacrine signalling, membrane-bound cytokines interact with the membrane-bound receptors of neighbouring cells to exert their effects. Cytokines can also act in a retrocrine manner in which soluble receptors bind to their corresponding membrane-bound cytokines to induce effects in the cytokine-expressing cell [25,26,27,28]. Under normal physiological conditions, most cytokines only exert their effects locally at secretion sites and are mainly stored in the extracellular matrix, while only trace levels of most cytokines are secreted into the bloodstream. Under certain pathological conditions, cytokines can act on distal cells in an endocrine manner to mediate systemic responses. Cytokines can be divided into two major classes based on their effect on inflammation, the first type, which are pro-inflammatory and can activate immune cells, and the second type, which are anti-inflammatory and can antagonize the effects of the first type cytokines. The interactions between these two classes jointly regulate immune activation and inactivation [25,26,27,28]. Many clinical characteristics of CSS can be explained by the effects of pro-inflammatory cytokines.

Although many cytokines are known to play a part in the development and progression of CSS, it is unclear which cytokine or which kind of cytokines are involved in CSS [2,3]. Accumulated studies have shown that IL-1β and TNF-α can promote the secretion of other cytokines, and are thus known as early phase cytokines. After stimulation by pathogenic factors, IL-1β and TNF-α are rapidly secreted, peaking after several hours. Subsequently, the body starts to secrete anti-inflammatory cytokines to regulate inflammatory responses and enable the body to counteract stimulation induced by harmful pathogens and maintain cellular homeostasis(Figure 1(B)) [29]. However, when there is continuous, strong stimulation from harmful pathogens or excessive immune responses, the equilibrium between pro- and anti-inflammatory responses is disrupted. These early phase cytokines can further promote the activation and release of cytokines and chemokines such as IL-2, IL-6, IL-8, IL-12, MIP-1α, and MIP-1β to cause a cascade-like reaction, thereby resulting in uncontrollable inflammatory responses [7]. Importantly, although HLH/CSS and severe sepsis patients can have high peripheral blood levels of cytokines, their cytokine expression spectra are completely different. During HLH/CSS, IFN-γ and IL-10 are significantly elevated, whereas IL-6 is only slightly elevated. In contrast, IL-6 and IL-10 are significantly elevated in sepsis, whereas IFN-γ levels are normal or only slightly elevated [14,15,16,17,18,19]. Different etiologies cause CSS through different mechanisms, and thus will induce changes in a different complement of cytokines. For example, the major cytokines involved in SARS in descending order are IL1β, IL6, IL12, IFNγ, IP10 (CXCL10), and MCP1, whereas the major cytokines in MERS in descending order are IFNγ, TNFα, IL15, and IL17. In COVID-19, blood levels of IL2, IL7, IL10, GSCF, IP10, MCP1, MIP1A, and TNFα are significantly higher in patients with severe illness than in those with non-severe illness (Table 1) [30,31,32,33,34,35,36,37]. The recent studies have shown that there is no significant difference in blood IL-6 levels between with mild and severe COVID-19 patients, suggesting that the clinical use of IL-6 antagonists may not be effective. These differences in the major cytokines is associated with differences in treatments and pathogenesis as well as individual clinical presentation and laboratory test results[31,38]. It should be emphasized that although cytokines and chemokines play important roles in the occurrence and progression of CSS, the concentrations of these cytokines and chemokines are not a basis for clinical diagnosis of CSS [13,14,15, 39].

**Table 1.** Major cytokines and chemokines involved in cytokine storm syndrome with different etiologies

Etiology	Cytokines and chemokines
CAR-T	IFN-γ, IL-2, IL-2Ra, IL-6, sIL-6R, GM-CSF, IL-1, IL-10, IL-12, TNF-a, IFN-a, MCP-1, MIP-1A
H5N1	MCP-1, CXCL10, CXCL9, IL-8
HIN1	IL-8, IL-9, IL-17, IL-6, TNF-α, IL-15, IL-12p70, IL-6
SARS-CoV-1	IL-1β, IL-6, IL-12, IFN-γ, IP10, MCP-1
MERS-CoV	IFN-γ, TNF-α, IL-15, IL-17
SARS-CoV-2	IL-2, IL-7, IL-10, G-SCF, IP10, MCP-1, MIP-1A, TNF-α

## Evolution of CSS diagnostic criteria

Because the clinical signs and symptoms of HLH/CSS are non-specific, clinicians must be particularly careful and attentive when making a diagnosis. The clinical presentation of CSS is similar to that of septic shock as well as the systemic presentation of other diseases, resulting in considerable difficulty in the clinical identification and diagnosis of CSS [14,15,16,17,18]. It is generally considered that disease onset before the age of 2 is due to pHLH/CSS, whereas most patients with disease onset after the age of 8 is due to sHLH/CSS. For patients with an age of onset between 2 and 8 years, the clinical presentation should be used as a basis for diagnosis. However, if ambiguity in identification remains, the diagnosis should be pHLH/CSS [14,15,16,17,18]. In 1991, in view of the variety of symptoms and laboratory tests for CSS, the international FHL Study Group put forth the first diagnostic criteria for HLH/CSS (HLH-1991) based on clinical, laboratory, and histopathological markers. These diagnostic criteria consist of five easily determined clinical and laboratory markers; however, they are not universal. Due to limitations in HLH/CSS knowledge at that time, the HLH-1991 diagnostic criteria are not applicable for many patients with atypical or insidious disease. Three years later, the International Histiocyte Society partially revised the HLH-1991 diagnostic criteria to generate the HLH-1994 diagnostic criteria, representing the first truly international HLH/CSS study. With continuous in-depth systemic research on HLH/CSS, particularly studies on the immunopathophysiological mechanisms of HLH/CSS, and the use of modern advanced diagnostic techniques, the International Histiocyte Society further revised the HLH-1994 diagnostic criteria, which were in use for 10 years as of 2004, to generate the HLH-2004 diagnostic criteria, etiological diagnosis, and HScore methods. These are currently the most accepted diagnostic tools for CSS [13,14,39,40,41,42].

According to the HLH-2004 diagnostic criteria, patients must fulfil any one of the following two criteria to be diagnosed with HLH/CSS [14,39]. The first HLH/CSS-related molecular biological diagnostic criteria is to have HLH/CSS-related pathogenic gene mutations among the following genes: PRF1, UNC13D, STX11, STXBP2, RAB27A, LYST, SH2D1A, BIRC4, ITK, AP3β1, MAGTI, CD27 and so on (Tables 2 and 3) [14]. The second criteria is to present with any five of the following eight symptoms: (1) a fever of >38.5°C that persists for >7 days; (2) splenomegaly; (3) cytopenia involving two or three lineages in the peripheral blood (hemoglobin <90 g/l, platelet count <100 × 10^9^/l, and/or neutrophil count <1.0 × 10^9^/l) that is not caused by reduced bone marrow hematopoiesis; (4) hyperglyceridemia and/or hypofibrinogenemia (triglycerides > 3 mmol/l or 3 standard deviations higher than the same age group, and fibrinogen ≤ 1.5 g/l or 3 standard deviations lower than the same age group); (5) presence of hemophagocytosis in the bone marrow, spleen, liver, or lymph nodes; (6) elevated serum ferritin levels (≥ 500 μg/l); (7) reduced NK cell activity or loss; and/or (8) elevated interleukin-2 receptor (sCD25) ≥ 2400 U/ml. Because molecular biological diagnosis and tests for NK cell activity and sCD25 are still not performed in many hospitals, and because a diagnosis requires meeting five out of the remaining six criteria, each criterion is particularly important (Tables 4 and 5) [14,18]. In addition, rapid and accurate diagnosis is critical due to the deadly nature of HLH/CSS. It should be noted that although the sensitivity and specificity for serum ferritin and sIL-2r are high, serum ferritin levels are also elevated when the body is in an inflamed state, during infection, or when the immune system is stimulated. Only when ferritin levels are greater than or equal to 10,000 mg/l will the specificity and sensitivity of HLH/CSS diagnosis be 96% and 90%, respectively. This suggests that as the disease progresses, significant serum ferritin elevation can be used for differential diagnosis of HLH/CSS [14,39].

**Table 2.** Gene Associated with fHLH/CSS.

	Syndrome	Gene and chromosome	OMIM	Protein name (bold) and functions	Clinical features	Inheritance
pHLH without hypopigmentation	pHLH1	Unknown 9q21.3-22	267700	Unknown	HLH	autosomal recessive
	pHLH2	*PRF1* 10q22.	603553	Unknown	Severe HLH, often with early onset	autosomal recessive
	pHLH3	*UNC13D* 17q25.1	608898	**Perforin** Pore forming protein, apoptosis, cytotoxicity	Severe HLH, often with early onset and/or CNS involvement	autosomal recessive
	pHLH4	*STX11* 6q24.2	603552	**Munc13-4** Vesicle priming factor	HLH, potentially with later onset & lower severity than pHLH2/3; congenital cardiac defects in some	autosomal recessive
	pHLH5	*STXBP2* 19p13.2	61310	**Munc 18-2/Syntaxin-binding protein** Vesicle fusion with cell membrane	Diarrhea & colitis, hypogammag- lobulinemia, sensorineural hearing loss, bleeding diatheses, neurologic abnormalities	autosomal recessive
pHLH with partial albinism	Griscelli syndrome	*RAB27A* 15q2.3	607624	**Rab27a** GTPase, promotes vesicle docking to the cell membrane	Recurrent HLH with waxing/waning phases and neurologic sequelae, hair with large irregular melanin granules	autosomal recessive
	ChediakHigashi	*LYST* 1q42.3	214500	**Lyst** Lysosomal trafficking, protein sorting	Giant lysosomes in leukocytes, pyogenic infections, gingival/ periodontal disease, bleeding, neurologic manifestations	autosomal recessive
	HermanskyPudlak 2	*AP3B1* 5q14.1	608233	**AP3βchain** Trafficking from Golgi to granules	Facial dysmorphisms, platelet defects &bleeding, neurologic manifestations, interstitial lung disease, colitis, neutropenia	autosomal recessive
pHLH with EBV- LPD	ITK deficiency	*ITK* 5q33.3	613011	**Itk** IL-2 inducible T cell kinase, T cell signaling	Progressive CD4 T cell loss and hypogammaglobulinemia, Hodgkin’s lymphoma	autosomal recessive
	CD27 deficiency	*CD27* 12p13.31	615122	**CD27** TNFR member, lymphocyte co-stimulation	Combined immunodeficiency with susceptibility to viral infections	autosomal recessive
	MAGT1 deficiency	*MAGT1* Xq21.1	300853	**Magnesium transporter 1** T cell activation via TCR	Combined immunodeficiency with loss of T cells, but elevations in CD19+ B cells	autosomal recessive
	XLP 1	*SH2D1A* Xq25	308240	**SAP** Activation of lymphocytes	Hypogammaglobulinemia, fulminant EBV infectious mononucleosis	X-linked
pHLH without EBV- LPD	XLP 2	*XIAP* Xq25	300635	**XIAP** Inhibition of apoptosis	Recurrent HLH, colitis	X-linked
	LPI	*SLC7A7* 14q11.2	222700	**SLC7A7** Amino acid transport	Gastrointestinal symptoms, Intellectual disability, hyperammonemia	autosomal recessive

Note: Table2 adapted from reference 14.

**Table 3.** Gene Associated with sHLH/CSS.

Gene function/pathway	Gene	Function	Gene function alteration	sHLH triggers
Granule-mediated cytolytic pathway	*PRF1*	Pore formation	Decreased/absent	infection, autoinflammatory, autoimmune, malignancy
	*UNC13D*	Granule priming	Decreased/absent	autoinflammatory, autoimmune, malignancy
	*STX11*	Granule fusion	Decreased/absent	autoinflammatory, malignancy
	*STXBP2*	Granule fusion	Decreased/absent	autoinflammatory, malignancy
	*Rab27a*	Granule docking	Decreased/absent	autoinflammatory,
	*LYST*	Granule trafficking	Decreased/absent	infection, autoinflammatory
Microtubule organization	*CCDC141*	Migration	Unknown	autoinflammatory
	*MICAL2*	Actin depolymerization	Unknown	autoinflammatory
	*ARHGAP21*	Regulates actin dynamics	Unknown	autoinflammatory
	*XIRP2*	Actin cytoskeleton stabilization	Unknown	infection, autoinflammatory
Cytokine production/signallling pathway Cytokine production/signalling pathway	*TGFB*	Immunoregulation	Unknown	infection
	*IFNGR1*	IFNγ receptor	Decreased/absent	infection
	*IFNGR2*	IFNγ receptor	Decreased/absent	infection
	*IL-10*	Immunoregulation	Unknown	infection
	*MEFV*	Pyrin inflammasome, cytokine production	Unknown	infection, autoimmune, autoinflammatory
	*NLRC4*	NOD-like receptor, cytokine production	Activated	autoinflammatory
	*IRF5*	Interferon regulatory factor transcription factor	Unknown	infection, autoimmune
	*CADPS2*	Regulation of exocytosis	Unknown	autoinflammatory
	*FKBPL*	Immunoregulation and cell cycle control	Unknown	autoinflammatory
	*GDI1*	Vesicular trafficking between organelles	Unknown	autoinflammatory
	*FAM160A2*	FTS/Hook/FHIP complex	Unknown	autoinflammatory
NK cell receptors	*KIR2DS5*	Immunoglobulin-like receptor	Unknown	infection
	*KIR3DS1*	Immunoglobulin-like receptor	Unknown	infection
Cell signalling	*ALK*	Receptor tyrosine kinase	Activating	malignancy
	*SH2D1A*	Signalling in T and NK cells	Decreased/absent	infection
	*XIAP*	Apoptotic suppressor protein	Decreased/absent	infection
	*CD27*	TNF-receptor superfamily	Decreased/absent	infection
	*CD70*	CD27 ligand	Decreased/absent	infection
	*MAGT1*	Magnesium transporter, N-glycosylation	Decreased/absent	infection
	*ITK*	Intracellular tyrosine kinase in T cells	Decreased/absent	infection, malignancy
Gene expression/transcriptional regulation	*GATA2*	Zinc-finger transcription factor	Decreased/absent	infection, malignancy
	*EZH2*	Maintaining transcriptional repression	Decreased/absent	malignancy
	*MYST3-CREBBP* fusion	Histone acetyltransferases	Activating	malignancy

Note: Table 3 adapted from Ref. [14].

**Table 4:** . Clinical and laboratory features of cytokine storm syndrome.

System	Clinical manifestations	Laboratory findings
General	Fever	Elevated C reactive protein
		Fall in erythrocyte sedimentation rate
		Elevated soluble interleukin 2 receptor
Hematological	Petechiae	Leukopenia
	Purpura	Anemia
	Ecchymoses	Thrombocytopenia
	Epistaxis	Hemophagocytosis in bone marrow aspiration
	Lymphadenopathy	Hyperferritinemia
Skin	Rash	∼
	Erythroderma	∼
	Edema	∼
Respiratory	Acute respiratory distress	∼
	Pulmonary infiltrates	∼
Renal	∼	Acute kidney injury
Gastrointestinal	Hematemesis	Transaminitis
	Rectal	Bleeding
	Hepatomegaly	Hypoalbuminemia
	Splenomegaly	Elevated
	∼	Elevated triglycerides
Central nervous system	Altered mental state	Pleiocytosis in cerebrospinal fluid
	Seizures	∼
	Encephalopathy	∼
	Coma	∼

Note: Table 4 adapted from Ref. [14].

**Table 5.** Possible distinguishing factors between primary and secondary HLH/CSS.

Possible distinguishing factors	Primary	Secondary
Lower age at disease presentation	√	∼
More frequent albinism/hypopigmentation	√	∼
More frequent or severe central nervous system involvemen	√	∼
Lower IL-1b levels	√	∼
Lower fibrinogen levels	√	∼
Higher bilirubin levels	√	∼
Lower C-reactive protein or No difference in C-reactive protein	√	∼
Higher total lymphocyte percentage	√	∼
Lower WBC, platelets, neutrophil counts	√	∼
Higher soluble CD25 levels	√	∼
Lower ferritin levels or No difference in ferritin	√	∼
Higher soluble CD25/ferritin ratio	√	∼
Lower lactate dehydrogenase levels	√	∼
Lower S100A12 levels	√	∼

Note: Table 5 adapted from Ref. [18].

For IAHS/CSS caused by pathogenic microorganisms, not only must the HLH-2004 diagnostic criteria be met but also there should be evidence for an etiological diagnosis. Common etiological diagnoses include: viral infection, as demonstrated by serum viral antibodies or positive DNA and/or nucleic acid tests; and bacterial or fungal infection, as demonstrated by isolation of the pathogen from peripheral blood or bone marrow [14,39]. Although the specificity of the HScore method in sHLH/CSS diagnosis is high, specific markers are lacking. Notable, the HLH-2004 diagnostic criteria are not entirely suitable for HLH/CSS diagnosis of adult patients, as severe infections – such as systemic inflammatory response syndrome (SIRS), MODS, and macrophage activation syndrome – can satisfy the diagnostic criteria, making differential diagnosis difficult [40,41,42]. pHLH/CSS and sHLH/CSS can both be induced by infection or other immune-related factors. Hence, clinical differentiation of pHLH/CSS and sHLH/CSS does not significantly affect patient diagnosis. In addition, hemophagocytosis may be absent in the early stage of the disease in some HLH/CSS patients, and thus would not satisfy the HLH-2004 diagnostic criteria, which in turn can lead to a missed or incorrect diagnosis [13,14,39,40,41,42].

Due to the limitations and insufficiencies of the HLH-2004 diagnostic criteria, in 2009 the International Histiocyte Society announced new diagnostic criteria HLH-2009[13,14,40]. These criteria are simpler, faster, more specific, and easier to master in clinical practice. The criteria include: (1) molecular biology diagnosis of HLH/CSS or X-linked lymphoproliferative syndrome; (2) at least three out of the following four clinical presentations: fever, cytopenia (at least two lineages), splenomegaly, and/or hepatitis; (3) laboratory tests fulfilling at least one of the following four criteria: presence of hemophagocyte, sIL-2R elevation, serum ferritin elevation, and/or either loss of or significant decrease in NK cell function; and (4) other markers supporting HLH/CSS diagnosis, such as hypertriglyceridemia, hypofibrinogenemia, and hyponatremia. The HLH-2009 diagnostic criteria are based on several non-specific clinical presentations, thereby allowing SIRS, MODS, and severe sepsis to meet the diagnostic criteria. These diseases may cause fever, pancytopenia, hyperglyceridemia, hypofibrinogenemia, elevated ferritin levels, and hemophagocytosis, and even sIL-2R elevation. Therefore, patients with severe infection have a risk of undergoing chemotherapy, which will further suppress the immune system [13,14].

It should be emphasized that different from previous understanding of other diseases-associated CSS, COVID-19-associated CSS has not only unique epidemiological, virological, and clinical characteristics, but also laboratory parameters, and pathophysiology In particular, accumulating data have indicated that internationally recognized diagnostic criteria such the HLH-2004, HLH-2009, and the HScore method did not effectively identify COVID-19-associated CSS. This is an important discovery that deserves further study. For these reasons, Temple University COVID-19 Research Group recently design a new early predictive criteria for special diagnosis of COVID-19-associated CSS, by which it can be readily used in clinical practice to determine the need for an early therapeutic regimen, block the hyperimmune response and possibly decrease mortality^,[^43].

## Importance of the correct identification of hemophagocytes in diagnosing CSS

**Figure 2.**
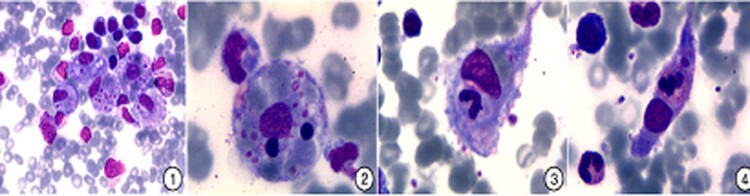
Morphological characteristics of hemophagocytes. (1) Focal distribution of hemophagocytes; (2) The hemophagocytes are round, which phagocytose red blood cells, lymphocytes and platelets; (3) The hemophagocytes are tadpole-like, which phagocytose red blood cells, neutrophilic stab granulocyte and platelets; and (4) The hemophagocytes are spindle-shaped, which phagocytose neutrophilic segmented granulocytes. Figure adapted from Ref. [44].

The first version of the HLH diagnostic criteria set in 1991 as well as the revised editions in 1994, 2004, and 2009 all considered hemophagocytosis as one of the most important criteria. HLH/CSS diagnostic criteria were proposed from the perspective of bone marrow cell morphology, of which the most important characteristic is the appearance of benign hyperplasia of mature tissue macrophages in bone marrow smears, accompanied by active hemophagocytes [13,14,39,40]. Hemophagocytes are a unique type of macrophage that engulfs blood cells with intact morphology and identifiable cellular structures and cell types through a process known as hemophagocytosis. Hemophagocytes that have phagocytosed either a single type of blood cell or two or more blood cells can be detected, and mature erythrocytes and platelets are the most common phagocytosed cell types (Figure 2) [44]. Hemophagocytes can be present in the bone marrow, liver, spleen, lymph nodes, central nervous system, and even in pleural effusion, ascites, and cerebrospinal fluid. Hemophagocytes are unevenly distributed in bone marrow smears and show both diffuse and focal distributions. In particular, hemophagocytes are concentrated at the tail of smears. Therefore, the entire slide should be searched for hemophagocytes. In early HLH/CSS, hemophagocytes are smaller in size and they only phagocytose a few cells, making them easily overlooked, resulting in a missed diagnosis and delayed treatment. Therefore, extra care should be given to prevent immature erythroblastic islands and macrophages that have phagocytosed cell debris from being misidentified as hemophagocytes [13,14,39,40].

Generally, hemophagocytes can be observed in the bone marrow of 25–100% of patients with a definitive diagnosis of HLH/CSS [45]. The reason for this difference may be due to the stage of the disease, sampling site, sample satisfaction, cell morphology testing capabilities of the medical institution, area and angle of the materials collected by the investigator, and population. Hemophagocytes were only detected in some patients with infection-related HLH/CSS when reviewing bone marrow smears. In some HLH/CSS patients, hemophagocytes can only be found following nucleated cell classification and enumeration in bone marrow, and hemophagocytes of different sizes and morphologies can be found at the head, surrounding region, and tail of the smear. Therefore, when a myelogram is conducted due to suspected HLH/CSS or fever, attention should be paid to the presence of hemophagocytes, particularly from multiple bone marrow aspiration sites when necessary. In one study of 32 pediatric patients with virus-related HLH/CSS, despite hemophagocytes being observed in all patients, they were only found in the second or third bone marrow examination in five patients and in the sixth bone marrow examination in one patient. The bone marrow of all hematologic malignancy patients with advanced HLH/CSS contained hemophagocytes; however, hemophagocytes were only found in 59% of the initial marrow samples [46]. Some studies found that these classical hemophagocytes are usually not seen in bone marrow at the early stages of disease, and the function of these hemophagocytes is established during disease progression [47]. At the early stages of virus-related HLH/CSS, the myelogram may appear normal, and hemophagocytosis may be absent. However, as the disease progresses, nucleated cell proliferation decreases, and hemophagocytes subsequently appears and gradually increases, accompanied by active hemophagocytosis. Accumulated studies have indicated that the sensitivity of hemophagocytosis in HLH/CSS diagnosis is around 60% [48]. Another small-scale case control study found that the sensitivity and specificity of hemophagocytes in HLH/CSS diagnosis are 83% and 60%, respectively. Bone marrow aspiration and biopsy can aid in determining the cause of cytopenia and exclude infection or malignancy. However, a diagnosis of HLH/CSS does not mean that hemophagocytes must be present in bone marrow aspiration, as its sensitivity is only 60–85%. Hemophagocytes can also be observed during infection, after blood transfusion, in other autoimmune diseases, and in bone marrow failure. In addition, some conditions may also cause false negatives, such as the early stage of disease, use of immunosuppressants, or recent blood transfusion [49,50].

It should be noted that hemophagocytes are only one of the eight diagnostic criteria established in HLH-2004 and one of the four criteria from HLH-2009 [5]. Therefore, HLH/CSS cannot be ruled out if hemophagocytes are not observed. Conversely, the presence of both hemophagocytes and peripheral blood cytopenia in two or more lineages is not sufficient for a definitive diagnosis of HLH/CSS. Hemophagocytosis is not an HLH/CSS-specific laboratory marker. The role and function of hemophagocytosis in HLH/CSS diagnosis should be correctly determined, and diagnosing HLH/CSS based solely on the identification of hemophagocytes would be inaccurate. Rather, a definitive diagnosis can be obtained only when the HLH-2004 or HLH-2009 diagnostic criteria are met [3,13,14,39]. However, observation of hemophagocytes raises the possibility of a clinical diagnosis of HLH/CSS and facilitates a timely diagnosis or exclusion of HLH/CSS. For patients with lymphoma-associated HLH/CSS, the many overlaps in clinical characteristics between lymphoma and HLH/CSS make diagnosing comorbid HLH/CSS difficult. However, the presence of hemophagocytosis in lymphoma patients strongly suggests the presence of HLH/CSS.

In summary, HLH/CSS has diverse etiologies, complex pathogeneses, and varying clinical presentations. In-depth research on the pathogenesis of HLH/CSS over the last 20 years has greatly increased the number of patients that were able to be definitively diagnosed with HLH/CSS. It should be emphasized that HLH/CSS diagnosis is difficult at the early stages due to the non-specific clinical signs and symptoms, which tends to result in missed and incorrect diagnoses. As HLH/CSS progresses rapidly and has a high mortality rate, quick and rapid diagnosis by clinicians is essential for patient survival. Therefore, clinicians should not only possess extensive clinical experience to ensure high sensitivity to the characteristics of HLH/CSS but must also be familiar with the HLH-2004/2009 diagnostic criteria, etiological diagnosis, and HScore methods. In addition, clinicians must also understand that there are some limitations to these diagnostic criteria. Therefore, it is extremely important that we continue to explore and discover early diagnostic markers of HLH/CSS with higher specificity and sensitivity.
